# Adherence to the Mediterranean Diet and Its Association With Sleep Quality and Chronotype Among Youth: A Cross-Sectional Study

**DOI:** 10.3389/fnut.2021.805955

**Published:** 2022-01-19

**Authors:** Farah Naja, Hayder Hasan, Safiya Hassan Khadem, Maryam Ahmed Buanq, Haya Khalid Al-Mulla, Aysha Khalifa Aljassmi, MoezAlIslam Ezzat Faris

**Affiliations:** Department of Clinical Nutrition and Dietetics, College of Health Sciences, Research Institute for Medical and Health Sciences (RIMHS), University of Sharjah, Sharjah, United Arab Emirates

**Keywords:** healthy diet adherence, chrononutrition, chronobiology, circadian and sleep physiology, sleep quality, circadian rhythm

## Abstract

**Background:**

Evidence indicates that many university students have poor adherence to a healthy diet accompanied by unhealthy lifestyle behaviors. Chrono-nutrition is an emerging field of research that examines the pattern of optimum daily activity in relation to the human's dietary patterns, and their reflections of variable health indicators such as sleep quality. However, there is a scarcity of research that examines the relationship between adherence to the healthy eating pattern, like the Mediterranean diet (MD), with sleep quality and chronotype among university students.

**Methods:**

A cross-sectional study was conducted, and convenience sampling was used. Participants were assessed for adherence to the MD using the Mediterranean Diet Quality Index (KIDMED), for sleep quality using the Pittsburgh Sleep Quality Index (PSQI), and for chronotype using the Morningness-Eveningness questionnaire (MEQ).

**Results:**

The study included 503 university students, most of them (81.5%) were females. Only 15.1 and 16.9% reported morningness chronotype and good sleep quality, respectively. About half of the students showed medium and high adherence to the KIMED. In-depth analysis revealed that students with good adherence to the MD were more likely to have a good sleep quality (OR = 0.35; 95%CI: 0.21–0.59; *P* < 0.001) even after adjustment for age and sex (OR = 0.36; 95%CI: 0.21–0.62; *P* < 0.001). The regression analysis also showed that those with good adherence to the MD had a significant association with better subjective sleep quality, less sleep latency, sleep disturbance, and daytime dysfunction even after adjustment for age and sex. Those with morningness chronotype had about a six-fold higher chance to have good adherence to the MD (OR = 5.67; 95%CI: 2.86–11.26; *P* < 0.001, respectively).

**Conclusions:**

Good adherence to the healthy diet presented in the MD among university students is associated with morningness chronotype and with improved overall sleep quality and sleep components. Long-term, controlled intervention research works are warranted for more elaboration on the impact of chronotype and dietary habits on sleep quality and other important aspects such as mental health and academic achievement.

## Introduction

The Mediterranean diet (MD), native to countries surrounding the Mediterranean basin, has been consistently associated with improved individual health (1, 2). The MD is characterized by increased consumption of a wide variety of local and seasonal fruits and vegetables, whole grains, moderate amounts of dairy products, plant-based protein sources, with olive oil and olives being the main source of fat. In addition, MD contains limited amounts of red meat. Within this diet, salt is partially replaced by the use of spices and herbs (3). The MD has recently been described as a lifestyle pattern that extends beyond dietary intake where regular physical activity, sharing meals with other people, and enjoying life are all part of this pattern (4). Research on MD indicates that it is rich in vitamins, fibers, probiotics, monounsaturated fatty acids (MUFA) and polyunsaturated fatty acids (PUFA), bioactive antioxidants and low glycemic foods, as well as anti-inflammatory agents; all of which contribute to impacting better lipid profile and reducing obesity and the associated inflammatory state, metabolic syndrome, type 2 diabetes mellitus (T2DM) and cardiovascular disease (CVD) (5–10). Several physiological mechanisms have been suggested to mediate the effect of the MD on health including microbiome diversity, lowering low-density lipoproteins (LDL) levels, oxidative stress, and inflammation, and an improvement in immune function and insulin sensitivity (6). In addition to these physiological mechanisms, higher adherence to the MD has been associated with lower c-reactive protein (CRP) and inflammatory cytokines secretion, including interleukin-1 (IL-1), tumor necrosis factor-α, and IL-6 (11). Increased levels of these inflammatory markers have been linked with poor sleep quality among different groups of people (12, 13).

Sleep, an important dimension, and determinant of lifestyle is a complex homeostatic process that involves multiple processes, including sleep-wake homeostasis, circadian cycle, and human dietary and lifestyle behaviors (14). Adequate sleep quality plays a pivotal role in health and exerts a positive influence on psychomotor performance, neurocognitive functioning, and physical health (15). Globally, reduced sleep quality is increasing at an alarming rate in the current era (16), mostly due to the excessive use of the blue screen especially at night times (17). Poor sleep quality has been associated with increased risks for obesity, T2DM, and CVD (18).

Related to sleep is the chronotype, defined as the expression of an individual's diurnal preferences in experiencing various psychological and biological traits including the wakefulness and timing of sleep (19). Individuals who sleep and wake up early and perform daily activities optimally during the morning hours are classified as having a morningness preference or are known as morning-type. In contrast, those who sleep and wake up late, ideally perform their daily activities in the evening hours are classified as having an eveningness preference or are recognized as evening-type. The intermediate type refers to individuals who can be either morning or evening type. The chronotype is a characteristic of the circadian timing system that presents differences between sexes and is affected by geographical factors such as latitude (20, 21). Chronotype has been shown to modulate sleep quality, sleep duration, and social jet lag in shift-workers (22). This notion supports the relationship between chronotype and sleep quality, and strengthens the hypothesis that sleep quality is better in morning types than evening types due to the social jetlag often experienced by the people with evening chronotype (23, 24).

A growing body of evidence signifies the relationship between diet and sleep, with several foods and food components that have been found to improve sleep duration and quality. Among these, tryptophan, and foods containing tryptophan, melatonin, and phytonutrients were looked at as sleep-promoting foods linked to improved sleep outcomes (25, 26), with those foods triggering the synthesis of serotonin and melatonin counted among the most helpful in promoting sleep (27). Further, dietary factors can profoundly impact the inflammatory status and hormones that can either directly or indirectly contribute to poor sleep quality, with variable effects for the major nutritional factors such as lipids, carbohydrates, and vitamins on sleep and sleep disorders (28).

While the MD and adequate sleep quality are important determinants of health throughout the lifecycle, they are of utmost importance among youth and adolescents. Youth and young adulthood is a period between 15 and 25 years of age, characterized by dramatic psychological and physiological changes, such as increased levels of autonomy in decision-making and increased self-development and identity (29). People at that age tend to have a nutrient-poor diet, often accompanied by high consumption of sugar-sweetened beverages and other energy-dense fast food (30). Among youth and adolescents, college students are particularly vulnerable to poor dietary habits because they have to adapt to new lifestyle changes associated with increased independence, time-consuming and hectic schedules, and high academic demands and responsibilities (31). These factors together were associated with increased consumption of caffeine, energy drinks (32, 33), and fast foods (34) by university students. University students' diets were shown to be low in vegetables and fruits, and high in sodium, carbohydrates, fats, and, making them energy-dense yet nutrient-poor options (35). University students in the United Arab Emirates (UAE) have been found to eat unplanned meals consisting primarily of unhealthy, nutrient-poor, and energy-dense foods (36). Furthermore, university students in the UAE have been observed to consume a lot of soft drinks and fast food, as well as skipping meals (34, 37). The negative health implication of such poor dietary habits among university and college students are further exacerbated by suboptimal sleep quality. Studies have shown that this population group is at high risk for sleep disorders (38). A review of the literature revealed that 55% of college students in the Gulf Cooperation Council (GCC) have irregular sleep schedules and get <7 h of sleep per night (39). While the relationship between adherence to the MD and sleep quality has been the subject of intensive research, little is known about the association between adherence to the MD and chronotype among university students. Thus, this study aimed primarily to (1) Examine the degree of adherence to the MD, sleep quality, and chronotype among youth, (2) Investigate the relationship between adherence to the MD on sleep quality, and (3) Explore the relationship between chronotype and adherence to the MD, and finally, (4) to explore whether there is an association between sleep quality and chronotype.

## Methods

### Study Design and Participants

To achieve the study objectives, a cross-sectional survey was conducted among students at the University of Sharjah (UoS), Sharjah, UAE. The UoS is a national university that has a diverse study body, coming from various ethnicities and backgrounds. Data collection was carried out between January and May 2021, using a convenience sampling approach. An email inviting students to take part in the study was sent by the central administration to all students. Students, both males, and females, aged 18 years and above and registered at the university during the time of data collection were eligible to participate in this study. The protocol of this study was designed and implemented in line with the guidelines specified by the Declaration of Helsinki. The survey's protocol and data collection instrument were reviewed and approved by the Research Ethics Committee at the UoS (REC-21-03-03-09-S). Before the start of data collection, participants were presented with a brief introduction about the study, its objectives, and procedure, and were asked to provide a signed electronic informed consent, in case they agreed to participate. Participants were informed that their participation is anonymous and completely voluntary in that they can choose or not to answer any particular question and that they are free to withdraw from the study at any time. Participation was completely voluntary, with no monetary or non-monetary incentives were given to the study participants.

Sample size calculations showed that 479 students are needed to estimate a 13.3% prevalence of good adherence to the MD with a power of 80% and an error margin of 3% (40). The 13.3% prevalence estimate used in the sample size calculations in this study was based on the results of a previous investigation of adherence levels to the MD among adolescents, conducted in Lebanon, a neighboring country of the Middle East and North African Region (41). The sample was representative of the students' population of the UoS as it included the same proportion of males to females (1:4) at the UoS, the majority of the students were UAE nationals and/or GCC citizens. Likewise, the medical and health sciences colleges were 4 out of 14 colleges, which is reflected by the number of participants from different college majors (29.6% medical and health sciences and 70.4% from other colleges).

### Data Collection Tool

For the data collection, a multicomponent, self-administrated online questionnaire was designed using Google Forms in both the English and Arabic languages. The link for the questionnaire was included within the body of the emails distributed to the participants. A panel of experts, consisting of a clinical nutritionist, a nutrition epidemiologist, and a public health scientist, developed the questionnaire choosing the scales for each of the concepts under study and ensuring its content validity. The questionnaire was first developed in the English language, later translated into Arabic, and back-translated into English. The original and back-translated versions of the questionnaire were compared for consistency and to ensure parallel-form reliability. Following the development of the questionnaire, a pilot test was conducted on 15 students to ensure cultural and context adaptability. The results of the pilot test were not included in the data for this study.

The questionnaire consisted of four main sections that addressed (1) sociodemographic characteristics, 2) adherence to the Mediterranean diet, (3) sleep quality, and (4) sleep chronotype. Except for the first section, the questionnaire referred to the month prior to the participation in the study. The average duration for the completion of the questionnaire was 10 min.

Section Introduction included questions to provide the following information about the participants: age (in years), sex (male; female), nationality (GCC-; non-GCC), marital status (single, married), income (low: <5,000 United Arab Emirates dirham [AED]; medium: 5,000–10,000 AED and high: >10,000 AED), smoking status (non-smoker, including past smoker, smoker) years spent at the university (<4, ≥4years), cumulative grade point average (CGPA) ( ≤ 3, >3), and major of studies at the university (health-related, non-health-related). In addition, participants were asked to report their weight (kg) and height (cm). Accordingly, the participant's body mass index (BMI) was calculated as weight (kg)/[height (m)]^2^.

The second section of the questionnaire examined adherence to the MD. For that purpose, The Mediterranean Diet Quality Index (KIDMED) was used. A systematic review of tools used showed that the KIDMED is the most widely used index to assess adherence to the MD among youth (42). The reliability of this index was found at *r* = 0.60 among college students (43). This index, first developed by Serra-Majem et al. (44), addressed food habits specific to the Mediterranean region. One of the important advantages of the KIDMED is that it can be self-administered as well as filled by an interviewer. The KIDMED consisted of 16 questions to which the answers could be either yes or no. Twelve of those questions were related to positive food habits and were related to the consumption of 1-fruits or fruit juice (daily), 2-second serving of fruit (daily), fresh or cooked vegetables (daily), fresh or cooked vegetables (more than once per day) fish (at least 2–3 times per week), legumes (more than once per week), pasta or rice (≥5 times per week), nuts (at least 2–3 times per week), use of olive oil at home, dairy products (2 yogurts and/or 40 g cheese daily), including cereals or grains for breakfast, and dairy for breakfast. For these positive food habits, the participants obtained 1 point for answering yes and 0 points for answering no, toward their KIDMED score. The remaining four questions, addressed negative food habits, consisting of skipping breakfast, including commercially baked goods or pastries for breakfast, sweets, and candy several times a day, and going to a fast-food restaurant more than once a week. For each of these four questions, the participant received a −1 if his/her answer was Yes and 0 if his/her answer was No. The sum of the points for all the 16 questions was calculated into a KIDMED score, ranging from (−3) to (+12), with higher scores indicating better adherence. Accordingly participating were grouped into three categories: good adherence (≥8), average (between 4 and 7), and poor ( ≤ 3) (44).

The third section of the questionnaire addressed the sleep quality among study participants (45). The Arabic version of the Pittsburgh Sleep Quality Index (PSQI) was used. The Arabic version of the PSQI was previously investigated for its validity and reliability and was found to be appropriate for use among Arabic-speaking natives (46). The PSQI is a self-rated questionnaire consisting of 19 questions addressing seven components/domains of sleep quality including subjective sleep quality, sleep latency, sleep duration, habitual sleep efficiency, sleep disturbances, use of sleeping medication, and daytime dysfunction. For each of those components, participants were assigned a score of “0–3,” whereby “0” indicates good, and “3” indicates “worst'. An overall PSQI score for sleep quality is generated as the sum of scores for the 7 components. As such, the overall PSQI ranged between 0 and 21 with higher scores indicating worse sleep quality. Using the overall PSQI score, participants were grouped as either having good ( ≤ 5) or poor (>5) sleep quality.

The last section of the questionnaire examined the participants' sleep chronotype (diurnal preferences) using the Morningness-Eveningness Questionnaire (MEQ), first developed by Horne and Östberg (47). A review of instruments used to evaluate chronotype indicated that the MEQ has been the gold standard addressing both sleep-wake information and appetite/exercise preferences while taking into account both psychological and behavioral factors when evaluating chronotypes (48). The MEQ consisted of 19 questions addressing personal daily sleep-wake habits and the times of day of preference of certain activities. Most of these questions are preferential whereby participants are asked to indicate when, for example, he/she would prefer to wake up or start sleep, rather than when he/she does. The MEQ questions are multiple-choice in nature, with each answer being assigned a specific value. The sum of these values results in an overall score ranging from 16 to 86 which can be categorized as follows: Definitely morning type 70–86, Moderately morning type 59-69, Neither type 42–58, Moderately evening type 31–41, Definitely evening type 16–30. The validity of the Arabic version of the MEQ was previously established and the results indicated that this version of the questionnaire provided valid and reliable measures of sleep chronotype (49).

### Data Analysis

Data analyses for this study were carried out using the Statistical Package for Social Sciences (SPSS) for Mac HD version 23 (SPSS Inc., Chicago, IL, USA). Participants' characteristics were described as mean (standard deviation), and frequency and proportions, for continuous and categorical variables, respectively. The characteristics were presented for the overall sample and by adherence to the MD. For the latter, participants were grouped, as per the KIDMED index, into “low”, “medium”, and “high” adherence. Comparisons of participants' characteristics between the poor and high adherence to the KIDMED were carried out using independent student's *t*-test for continuous variable and Chi-square tests for categorical variables. In order to investigate the effect of adherence to the MD on sleep quality, simple and multiple logistic regressions analyses were carried out, with the adherence to the MD (high vs. low) as the independent variable and sleep quality indicators as dependent variables. Sleep quality indicators consisted of the overall PSQI and its seven components. The effect of sleep chronotype on adherence to the MD was also examined using simple and multiple logistic regression analyses. For these analyses, the three classes of adherence to the MD were re-categorized into two categories (medium and high as one “High adherence,” and “Low adherence”) and considered as dependent variables, while the five chronotypes were re-categorized into three categories (eveningness, intermediate, and morningness) and entered as the independent variables. For all analyses, variables that showed significant associations with adherence to the MD in the univariate analyses were included in the multiple regression models. Results of the logistic regression analyses were presented as odds ratio (OR) and their corresponding 95% confidence interval (CI). A *p*-value <0.05 was considered statistically significant.

## Results

A total of 503 participants (81.5% were females) with a mean age of 22.11(4.2) years and a BMI of 23.97(2.09) kg/m^2^ and about one third were overweight/obese (32.6%). The majority were GCC nationals (88.3%) and single (87.7%), whereas about half of them had low income (51.5%) and 52.7% were in their first-third year of study. More than half of the students (56.5%) had a CGPA of >3/4. The vast majority were non-smokers (92.8%) and from non-medical and health sciences colleges (70.4%). Only the minority had morningness chronotype and a good PSQI (15.1 and 16.9%, respectively). About 54% (272/503) of the students showed medium and high adherence to the KIMED (Table 1).

**Table 1:** Sociodemographic characteristics of the participants (*n* = 503).

**Variables**	***n* (%)**	**Adherence to Mediterranean diet (KIDMED)**		***P-*value**
		**Low** ***n* = 231 (45.9%)**	High^**^ ***n* = 272 (54.1%)**	
Age (Years)^*^	22.11(4.2)	21.58(3.5)	22.57(4.67)	0.008
**Sex**				
Female	410 (81.5)	180(43.9)	230(56.1)	0.056
Male	93 (18.5)	51(54.8)	42(45.2)	
**Nationality**				
GCC	444 (88.3)	207(46.6)	237(53.4)	0.38
Non-GCC Arab/Non-Arab	59 (11.7)	24(40.7)	35(59.3)	
**Body mass index (BMI, kg/m** ^**2**^ **)**				
Underweight/Normal weight	339(67.4)	156(46)	183(54)	0.95
Overweight/obese	164(32.6)	75(45.7)	54.3)	
**Marital status**				
Single	441 (87.7)	206(46.7)	235(53.3)	0.34
Married	62 (12.3)	25(40.3)	37(59.7)	
**Income in AED (*****N*** **=** **335)**				
Low (<5,000)^$^	259 (51.5)	127(49)	132(51)	0.44
Medium (5,000–10,000)^$^	48 (9.5)	20(41.7)	28(58.3)	
High (>10,000)^$^	28 (5.6)	11(39.3)	17(60.7)	
**Smoking status**				
Non-smoker (including past smokers)	467 (92.8)	215(46)	252(54)	0.85
Smoker	36 (7.2)	16(44.4)	20(55.6)	
**Years at the university**				
<4 years	265 (52.7)	127(47.9)	138(52.1)	0.34
≥4 years	238 (47.3)	104(43.7)	134(56.3)	
**CGPA**				
≤ 3	219 (43.5)	103(47)	116(53)	0.66
>3	284 (56.5)	128(45.1)	156(54.9)	
**College major**				
Medical and Health Sciences	149 (29.6)	70(47)	79(53)	0.76
Non-medical	354 (70.4)	161(45.5)	193(54.5)	

* *Mean (Standard Deviation, SD); BMI, Body mass index; GCC, Gulf Cooperation Council; CGPA, Cumulative Grade Point Average out of 4; BMI (in kg/m^2^) according to the WHO classification: <18.5 = underweight, 18.5–24.9 = Normal weight, 25–29.9 and ≥30 = Obese)*.

** *Medium and high were included as high*.

$ *1 United States dollar = 3.67 AED (United Arab Emirates Dirham)*.

Table 1 also demonstrates the comparisons of KIDMED adherence with different sociodemographic variables. Those with medium and high adherence to KIDMED were significantly older than those with low adherence [22.57(4.67) vs. 21.58(3.5) years] and females showed more adherence compared to males (56.1% vs. 45.2%, *P* = 0.056).

**Figure 1:**
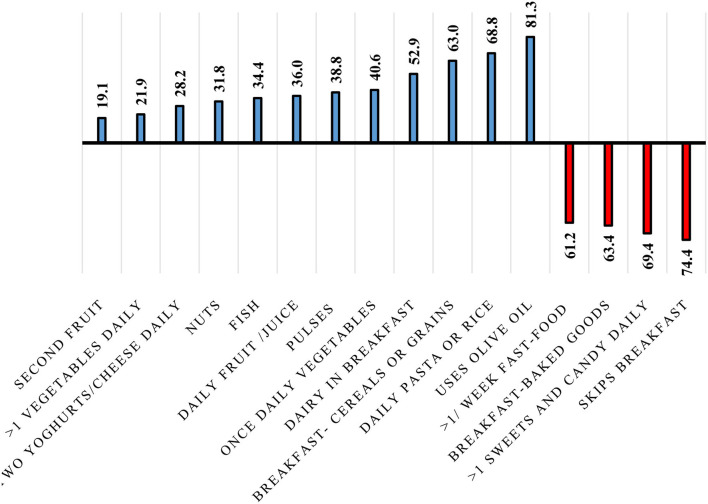
Prevalence of each of the 16 KIDMED components among the study participants (*n* = 503).

Figure 1 illustrates the prevalence of each KIDMED component. About one-fifth of the participants were having a second fruit every day and fresh or cooked vegetables more than once a day (19.1 and 21.9%, respectively). Additionally, more than half of the participants were consuming dairy products for breakfast (52.9%), cereals or grains for breakfast (63%), pasta or rice almost every day (68.8%), and using olive oil at home (81.3%). On the other hand, 61.2, 63.4, and 69.4% of the participants were going to a fast-food restaurant more than once a week, having commercially baked goods or pastries for breakfast, and taking sweets and candy several times every day, respectively. While three-quarters (74.4%) of the participants were skipping breakfast.

Table 2 demonstrates the association of the KIDMED with MEQ, PSQI, and PSQI components. Regarding chronotype, 80.3% of those with morningness chronotypes exhibited medium and high adherence to KIDMED compared to 39.8% for those with eveningness chronotypes (*P* < 0.001). Similarly, those with good PSQI (PSQI score ≤ 5) were about 3 times significantly (*P* < 0.001) more adherent to the KIDMED (74.1%) compared to those with poor PSQI (50%). In-depth analysis of the association of the KIDMED adherence with the PSQI components showed that those with good subjective sleep quality (60.2%), no sleep latency (60.5%), no sleep disturbance (57.4%), and no daytime dysfunction (60.5%) were significantly more adherent to medium and high KIDMED.

**Table 2:** Sleep quality and chronotype, overall and by the level of adherence to the MD, among study participants (*n* = 503).

**Variables**	***n* (%)**	**KIDMED**		*P-*value ^*^
		**Low** ***n =* 231 (45.9%)**	**Medium and High *n =* 272 (54.1%)**	
**MEQ^a^**				
Eveningness	123(24.5)	74(60.2)	49(39.8)	<0.001
Intermediate	304(60.4)	142(46.7)	162(53.3)	
Morningness	76(15.1)	15(19.7)	61(80.3)	
**Overall PSQI Score**				
Poor (>5)	418(83.1)	209(50)	209(50)	<0.001
Good (≤ 5)	85(16.9)	22(25.9)	63(74.1)	
**Components of the PSQI^b^**				
Subjective sleep quality				
Poor	161(32)	95(59)	66(41)	<0.001
Good	342(68)	136(39.8)	206(60.2)	
**Sleep latency**				
Poor	247(49.1)	130(52.6)	117(47.4)	0.003
Good	256(50.9)	101(39.5)	155(60.5)	
**Sleep duration**				
Poor	107(21.3)	57(53.3)	50(46.7)	0.08
Good	396(78.7)	174(73.9)	222(56.1)	
**Sleep efficiency**				
Poor	143(28.4)	71(49.7)	72(50.3)	0.29
Good	360(71.6)	160(44.4)	200(55.6)	
**Sleep disturbance**				
Poor	170(33.8)	89(52.4)	81(47.6)	0.04
Good	333(66.2)	142(42.6)	191(57.4)	
**Use of sleep medication**				
Poor	52(10.3)	25(48.1)	27(51.9)	0.74
Good	451(89.7)	206(45.7)	245(54.3)	
**Daytime dysfunction**				
Poor	161(32)	96(59.6)	65(40.4)	<0.001
Good	342(68)	135(39.5)	207(60.5)	

a *‘Morningness’ which includes ‘Definitely morning’ and ‘Moderately morning’, Intermediate, corresponding to ‘Neither morning nor evening’ and Eveningness consisting of ‘Definitely evening’ and ‘Moderately evening’*.

b *‘good’ corresponds to scores of 0 and 1; ‘poor’ corresponds to score 2 and 3 on each of the components*.

* *Using Chi-square test*.

**Figure 2:**
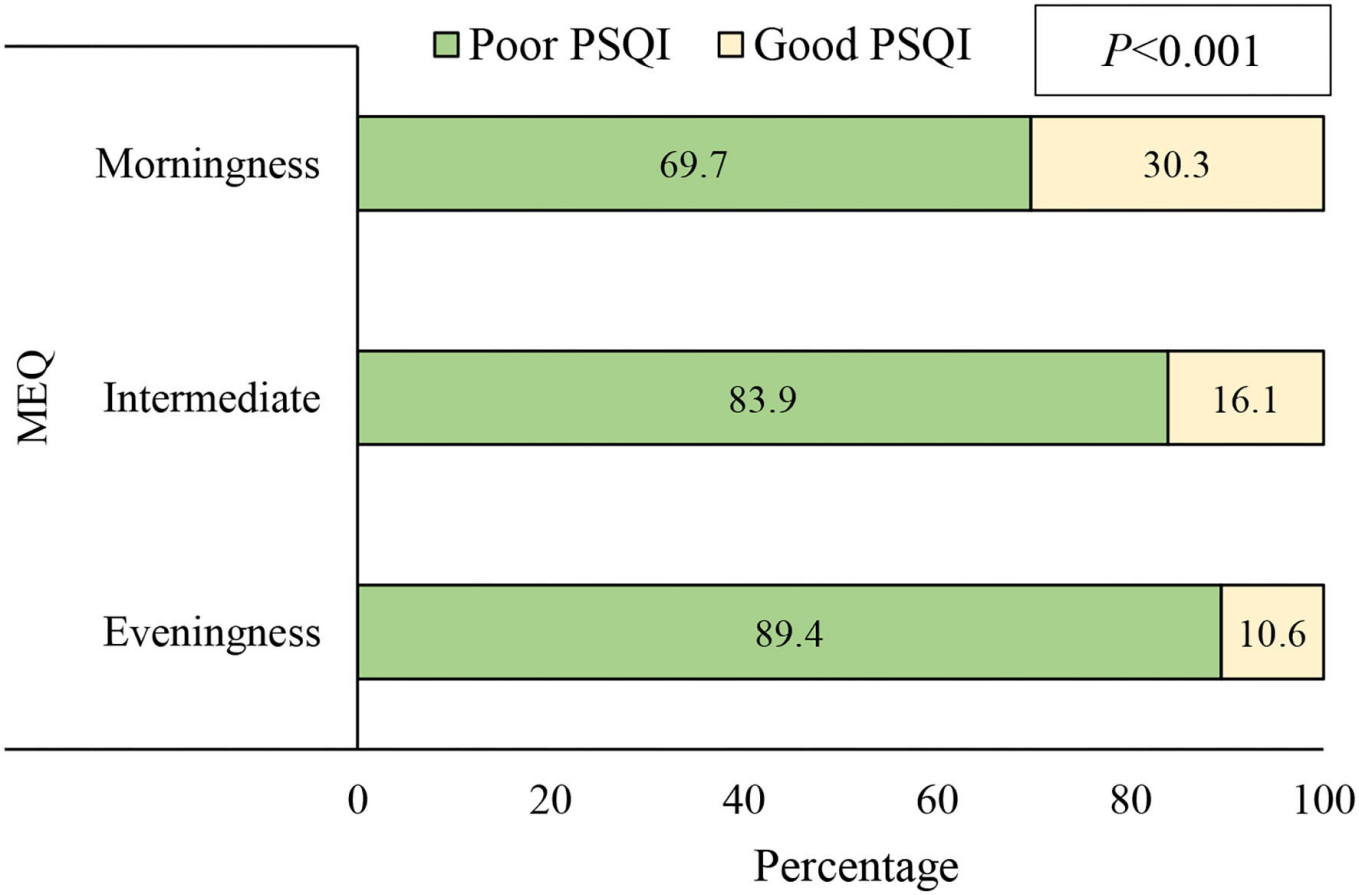
Association of the chronotype, as assessed by Morningness–Eveningness Questionnaire (MEQ), with sleep quality examined using Pittsburgh Sleep Quality Index (PSQI) among study participants (*n* = 503).

Figure 2 depicts that students with morningness chronotype were more likely to have good PSQI compared to those eveningness chronotype (30.3% vs. 10.6%, *P* < 0.001).

Table 3 shows the lack association of the overall PSQI and the KIDMED. Those with high KIDMED adherence had a significant association with subjective sleep quality (B = −0.78; OR = 0.48; 95% CI: 0.31–0.67; *P* < 0.001), sleep latency (B = −0.53; OR = 0.58; 95% CI: 0.41–0.83; *P* = 0.003), sleep disturbance (B = −0.39; OR = 0.67; 95% CI: 0.46–0.98; *P* = 0.04), and daytime dysfunction (B = −0.81; OR = 0.44; 95% CI: 0.30–0.64; *P* < 0.001) even after adjustment for age and sex.

**Table 3:** Simple and multiple logistic regression for the association of sleep quality with adherence to the Mediterranean diet among study participants (*n* = 503).

		***B***	**Unadjusted**				***B***	Adjusted^*^			
			**OR**	**95% CI**		***P*-value**		**OR**	**95% CI**		***P*-value**
				**Lower**	**Upper**				**Lower**	**Upper**	
Overall PSQI^**^											
KIDMED^***^	Low	Ref.	–	–	–	–	Ref.	–	–	–	–
	High	−0.05	0.94	0.59	1.51	0.81	−0.06	0.94	0.58	1.51	0.80
Subjective sleep quality^**^											
KIDMED	Low	Ref.	–	–	–	–	Ref.	–	–	–	–
	High	−0.78	0.46	0.31	0.67	**<0.001**	−0.69	0.50	0.34	0.73	**<0.001**
Sleep latency^**^											
KIDMED	Low	Ref.	–	–	–	–	Ref.	–	–	–	–
	High	−0.53	0.58	0.41	0.83	**0.003**	−0.55	0.58	0.40	0.83	**0.003**
Sleep duration^**^											
KIDMED	Low	Ref.	–	–	–	–	Ref.	–	–	–	–
	High	−0.37	0.69	0.45	1.05	0.08	−0.31	0.73	0.47	1.12	0.15
Sleep efficiency^**^											
KIDMED	Low	Ref.	–	–	–	–	Ref.	–	–	–	–
	High	−0.21	0.81	0.55	1.19	0.29	−0.20	0.81	0.54	1.20	0.30
Sleep disturbance^**^											
KIDMED	Low	Ref.	–	–	–	–	Ref.	–	–	–	–
	High	−0.39	0.67	0.46	0.98	**0.04**	−0.37	0.69	0.47	1.00	**0.05**
Use of sleep medication^**^											
KIDMED	Low	Ref.	–	–	–	–	Ref.	–	–	–	–
	High	−0.09	0.90	0.51	1.16	0.74	−0.08	0.91	0.51	1.64	0.77
Daytime dysfunction^**^											
KIDMED	Low	Ref.	–	–	–	–	Ref.	–	–	–	–
	High	−0.81	0.44	0.30	0.64	**<0.001**	−0.81	0.44	0.30	0.65	**<0.001**

*PSQI, Pittsburgh Sleep Quality Index; CI, Confidence Interval*.

* *Adjusted for age and sex*.

** *Dependent variable*.

*** *Independent variable*.

*Low is poor adherence to KIDMED and high indicates average and good adherence to KIDMED*.

Table 4 demonstrates the association of the KIDMED with the MEQ. Those with morningness chronotype had about six-fold higher chance to have medium and high KIDMED adherence (unadjusted OR = 6.14; 95%CI: 3.14–12; *P* <0.001 and adjusted OR = 5.67; 95%CI: 2.86–11.26; *P* <0.001, respectively).

**Table 4:** Logistic regression for the association of KIDMED with the chronotype among study participants (*n* = 503).

**Model**	**Independent variables**	**OR**	**KIDMED (Dependent variable)**		***p*-value**
			**95% CI**		
			**Lower**	**Upper**	
**Unadjusted**	**MEQ**				
	Eveningness	Ref			
	Intermediate	1.72	1.13	2.64	0.012
	Morningness	6.14	3.14	12.00	<0.001
**Adjusted**	**MEQ**				
	Eveningness	Ref			
	Intermediate	1.64	1.07	2.54	0.024
	Morningness	5.67	2.86	11.26	<0.001
	Age	1.05	1.00	1.11	0.035
	Sex	0.51	0.31	0.84	0.008

*MEQ, Morningness–Eveningness Questionnaire; CI, Confidence Interval*.

## Discussion

The current study is the first to examine the relationship between adherence to MD, subjectively measured sleep quality using PSQI, and the chronotype among university students. Findings of the current work showed that greater adherence to the MD is associated with better overall sleep quality and sleep components.

Sleep quality is strongly affected by food quantity and quality, with dietary inflammatory potential has been proposed as one of the inflecting factors that affect sleep quality and duration (50). Providing that lower inflammatory status has been consistently associated with improved sleep quality (51, 52), the well-established low antioxidant and anti-inflammatory potential of the MD (53) may help to explain, in part, the strong positive association between the good adherence to the MD and the reported good sleep quality components found among our study sample. The relationship between sleep and inflammation is controversial and bidirectional (12, 54, 55); that is, when the bodily inflammatory state is increased, the sleep quality is worsened, and when sleep quality is worsened, the inflammatory state becomes increased. The mechanism that elaborates such a relationship stems from the fact that inadequate sleep can increase the inflammatory response through increased cytokine secretion, including interleukin-1 (IL-1), tumor necrosis factor-α, and IL-6; the cytokines that are classically found to be associated with sleep deprivation (12). These pro-inflammatory cytokines are those consistently reported to be reduced in response to the long-term exposure and adherence to the MD, as revealed by many reports (11, 56).

Another underlying mechanism that explains the relationship between MD and improved sleep quality is the ability of the MD to increase melatonin secretion. Many fruits and vegetables are good sources of melatonin, and experimental studies reveal that consumption of these phytochemical-rich foods can improve sleep quality parameters and alleviate sleep disturbances (26, 57).

Further, the relationship between poor sleep quality and food intake has been consistently examined. Poor sleep quality and short sleep duration are associated with greater food intake and lower-quality diet, which are increasingly recognized as risk factors for obesity (58, 59). It was proposed that short sleep duration affects energy expenditure and/or energy intake in a way that creates a positive energy balance. Indeed, the sleep restriction triggers energy intake that exceeds the added energy costs of maintaining longer wakefulness (58).

From a health perspective, the morning chronotype has been the preferred chronotype over the evening one, with the former having less association with cardiometabolic risk factors and other health problems, including psychological, neurological, and gastrointestinal morbidities, and lower mortality rates compared to evening chronotypes (69). These health improvements associated with the morning chronotype could be also a mirror for the improved eating habits consistently reported with such chronotype, as revealed by the increased adherence to the MD by people with morning chronotype (70, 71). The reported low prevalence of morningness chronotype in comparison with the eveningness one (15.1% vs. 24.5%, respectively) among the study sample is expected, considering the frequently observed long stay up at night among surrounding and neighboring students, and in line with the low prevalence reported in other parts of the world. Similar distributions of morning chronotype (15.8 and 18.4%) were found among university students in Italy and China, respectively (72, 73). Such pattern of chronobiology among university students could be explained, in part, by the same aforementioned factors affecting their food selection and eating behaviors; that is the hectic schedule, high demanding and frequent academic tasks, and exams, with the students' persistent endeavor to catch the due dates and deadlines and to find less distracting times and more silent surrounding environment. Further, the excessive use of mobile applications and the massive reliance on blue screens for socialization play a role, in part, for such outcomes. Thirdly, the reported positive correlation between eveningness and higher cognitive ability [explaining why more people with higher cognitive ability are more likely to be nocturnal than others (74)] may explain the predominance of eveningness chronotype over other chronotypes among university students (73). Lastly, the frequently reported consumption of stimulant beverages such as caffeine-rich energy drinks and coffees (32, 33, 75) is among the fundamental factors for extending the wake night hours and shifting the chronotype into the eveningness pattern among university students. This finding of the predominance of eveningness pattern is consistent with the increased prevalence of sleep problems among university students, especially medical students, as revealed by our previously published systematic review and meta-analysis (39).

The relatively low prevalence of good sleep quality (about 17%) among our university students is also consistent with and mirrors for other reports in the UAE and other parts of the globe. However, this number is lower than previous estimates among students in the UAE, whereby the prevalence rates of good sleep, using similar assessment tools were 34% in 2020 and 44.8% in 2021, respectively (50, 76). Such a high heterogeneity in the reported prevalence of sleep quality among students in the same institute during two consecutive years is a mirror for the wide spectrum of interacting and interplaying variables impacting sleep quality, and the high sensitivity of sleep quality for variable environmental (dietary and lifestyle) factors. Parallel with the previous outcomes, the use of energy drinks among university students in the UoS was found to be significantly associated with reduced sleep quality, as revealed in our previous work in 2017 (32).

Having three-times higher reported good sleep quality among those with morningness chronotype than those with the evening one (30.3% vs. 10.6%), found in this study, is consistent with other reports among university students throughout the world, such as in Brazil (77, 78), India (79) and Korea (80), where eveningness chronotype was consistently associated with poorer sleep quality. Such a strong relationship is a reflection of the direct interaction between circadian rhythm and sleep in response to the chronotype (81). In a similar work among university students in Brazil, evening-style students with poor sleep quality presented shorter total sleep time, higher sleep latency, and late-onset of sleep; all imply a kind of lack of compatibility between the biological rhythms and the morning academic demands. Such incompatibility generates successive daily desynchronizations. Further, with morning academic duties, students with evening chronotype are more prone to be anxious in relation to their personality, which indicates a greater irregularity of the sleep-wake cycle than the other chronotypes, and puts them at higher risk of impairment of cognitive and psychological processes, social jet lag, and consequently, worse quality of life and academic performance and (78).

Age and sex are factors that are variably associated with the adherence to the MD (60), with conflicting results that have been found for the relationships with adherence to MD (61). Consistent with our findings, older age is one of the determining factors for the degree of adherence to the MD (60, 62). For instance, lower adherence to the MD during two decades from 1985 to 2005 in South Italy was found to be more pronounced in younger people than in older people (30–49 vs. 50–69 years) (62), while higher age was also associated with more adherence to the MD among patients with food neophobia in Italy (60). These findings are in line with the dominant nutrition transition taking place in many countries in the world, where traditional and healthier dietary patterns are slowly eroding and being replaced by “westernized” patterns of food consumption. This nutrition transition is most pronounced among the younger population, who are most susceptible to the globalization of food intake and the influence of fast food and processed food chains (63, 64).

Despite the well-known health-improving effects and disease-preventing impact of MD against chronic diseases, a low adherence rate to the MD has been repeatedly reported, especially in the Mediterranean countries themselves (61, 65), with sex has been identified as one of the multiple factors working as predictors of poor adherence to the MD (61). The superiority of females in reporting more adherence to the MD diet in our work is consistent with previous work showing that male personality traits were associated with low MD adherence among patients with ischemic heart disease (IHD) (66). However, female sex was shown to be more associated with low adherence in comparison to males in another study in Italy among adults with IHD (61), implying that various other factors such as age, health condition, cultural, demographic, and socioeconomic variables may play a role in determining the level of adherence to the MD.

Having fewer fruits than recommended, skipping breakfast, and frequently visiting fast-food restaurants reported among university students in our work is consistent with the repeatedly observed unhealthy behaviors among the university students in the UAE and other parts of the world (67, 68). University students' diets are low in fruits and vegetables and high in fats, carbohydrates, and sodium, making them energy-dense yet nutrient-poor options. In the UAE, university students have been found to eat unplanned meals consisting primarily of quick, unhealthy, energy-dense foods (36), and have been observed to consume a lot of fast food and soft drinks, as well as skipping meals (34, 37). Such unhealthy eating behaviors and poor food selections by university students have been ascribed to variable environmental factors that characterize the university academic life, such as the hectic schedule, high demanding and frequent academic tasks, and exams.

The current work entails some limitations that should be considered when interpreting the current findings. First, diet quantity was not examined in the current work, which may also affect sleep quality and may interact with different components of sleep. The inherent limitations of the cross-sectional design cannot be overridden in this context, where causality cannot be inferred in such an observational design. Other limitations related to the self-reported questionnaires such as recall bias, and response bias are worth to be kept in mind. Of particular importance is the self-reported nature of the assessment of sleep quality, which relies heavily on the participants' subjective assessment. Future studies could include objective measures of sleep quality, using polysomnography (82). In addition, this study explored only the associations between adherence to MD and sleep quality, not the mechanisms behind these associations. This study also does not account for possible confounding factors that may affect the relationship, such as evening thermogenic factors like nocturnal exercise, nocturnal light exposure, and spicy nocturnal meals, and physical activity level. Lastly, adjustments for the alpha value for multiple comparisons that were not performed need to be considered in future studies.

To conclude, good adherence to the MD among university students is more associated with morningness chronotype and improved overall sleep quality and sleep components. Improving the knowledge and attitude of the university students toward their dietary and lifestyle behaviors, and the significance of chronotype in determining their future disease risk factors are of pivotal importance. Further long-term, controlled intervention research works are warranted for more elaboration on the impact of chronotype and dietary habits on sleep quality.

## Data Availability Statement

The raw data supporting the conclusions of this article will be made available by the authors, without undue reservation.

## Ethics Statement

The studies involving human participants were reviewed and approved by University of Sharjah, REC approval no. (REC-21-03-03-09-S). The patients/participants provided their written informed consent to participate in this study.

## Author Contributions

MF: conceptualization, project administration, and funding acquisition. MF and FN: methodology, validation, supervision, and writing—original draft. HH: software. HH and FN: formal analysis and visualization. MF, FN, SK, MB, HA-M, and AA: investigation and data curation. MF, FN, and HH: resources and writing—review and editing. All authors contributed to the article and approved the submitted version.

## Conflict of Interest

The authors declare that the research was conducted in the absence of any commercial or financial relationships that could be construed as a potential conflict of interest.

## Publisher's Note

All claims expressed in this article are solely those of the authors and do not necessarily represent those of their affiliated organizations, or those of the publisher, the editors and the reviewers. Any product that may be evaluated in this article, or claim that may be made by its manufacturer, is not guaranteed or endorsed by the publisher.
